# Investigation of factors influencing inpatient antibiotic prescribing decisions in the Veterans’ Health Administration

**DOI:** 10.1017/ash.2022.230

**Published:** 2022-06-23

**Authors:** Evan T. Economos, Cassie Cunningham Goedken, Stacey Hockett Sherlock, Katie J. Suda, Matthew Goetz, Erin Balkenende, Emily E. Chasco, Aaron M. Scherer, Michihiko Goto, Eli N. Perencevich, Heather Schacht Reisinger, Daniel J. Livorsi, for the Veterans’ Affairs–Centers for Disease Control and Prevention Practice–Based Research Network (VA-CDC PBRN)

**Affiliations:** 1 Uiversity of Iowa Carver College of Medicine, Iowa City, Iowa; 2 Center for Access & Delivery Research & Evaluation (CADRE), Iowa City Veterans’ Affairs (VA) Health Care System, Iowa City, Iowa; 3 Center for Health Equity Research and Promotion, VA Pittsburgh Healthcare System, School of Medicine, University of Pittsburgh, Pittsburgh, Pennsylvania; 4 VA Greater Los Angeles Healthcare System, Los Angeles, California; 5 David Geffen School of Medicine, University of California–Los Angeles, Los Angeles, California; 6 Institute for Clinical and Translational Science, University of Iowa, Iowa City, Iowa

## Abstract

To investigate factors that influence antibiotic prescribing decisions, we interviewed 49 antibiotic stewardship champions and stakeholders across 15 hospitals. We conducted thematic analysis and subcoding of decisional factors. We identified 31 factors that influence antibiotic prescribing decisions. These factors may help stewardship programs identify educational targets and design more effective interventions.

Promoting antibiotic stewardship is important to combatting the spread of antibiotic-resistant bacteria, a public health threat. However, the implementation of antibiotic stewardship practices can be challenging because it involves changing providers’ knowledge, attitudes, and behaviors around antibiotic decision making.^1^ Few studies have assessed the range of factors that influence antibiotic decision making in acute-care settings. Understanding factors that influence these decisions can be an important prerequisite to identifying knowledge gaps and designing more effective antibiotic stewardship strategies. In this study, we identified factors that influence decision making about antibiotics across 15 hospitals in the Veterans’ Health Administration (VHA).

## Methods

During 2018–2019, the study team conducted semistructured interviews with 49 healthcare workers at 15 geographically dispersed VHA hospitals. Data on antibiotic use at these 15 hospitals have been published previously.^2^ These sites made up the Veterans’ Affairs–Centers for Disease Control and Prevention Practice–Based Research Network (VA-CDC PBRN). Each site had a dedicated research coordinator who was trained by the study team to conduct qualitative interviews.^3^

Interview participants included the antibiotic stewardship program physician and pharmacist champion at each of the 15 sites (n = 30), as well as other key stakeholders (n = 19) at 5 sites, including pharmacy administrators (n = 5), hospitalists (n = 5), ICU physicians (n = 5), and emergency department providers (n = 4). The interviews were designed to elicit perspectives on barriers and facilitators to the adoption of antibiotic management policies for fluoroquinolones and extended-spectrum cephalosporins, as previously described.^3^ Interviews also explored decisional factors that influence the use of any antibiotics for 4 common infections: pneumonia, urinary tract infections, intra-abdominal infections, and skin and soft-tissue infections. All interview guides can be found in the Supplementary Material.

Each interview was audio-recorded on encrypted recorders, when possible, and was then transcribed. Transcripts from 47 interviews and detailed notes from 2 interviews were uploaded into MAXQDA, a qualitative data management software program (VERBI Software, Berlin, Germany). An interdisciplinary team of physicians with antibiotic stewardship expertise and qualitative analysts analyzed the transcripts using thematic analysis, subcoding, and categorization.^4^ First, transcripts were reviewed by the analysis team, and a code book was developed from the interview guide and from emergent content. The transcripts were then coded by consensus.^5^ Second, the analysis team subcoded intersecting segments for “decisional factors” surrounding antibiotic prescribing for “pneumonia.” The final phase involved 2 team members (E.E. and D.L.) expanding the subcoding process to apply the decisional factor subcodes to interview segments addressing the other infection types. To be considered relevant for analysis, a decisional factor must have been mentioned by at least 2 participants. After all relevant factors were reviewed, team members discussed broader categories for potential grouping. Ultimately, each factor was grouped into 1 of 5 categories based on group consensus.

The Veterans’ Affairs Central Institutional Review Board granted a waiver for written informed consent for participants in the semistructured interviews. The study team reviewed but did not document informed consent information with all participants.

## Results

Table 1 provides an overview of the 15 participating hospitals. A common theme across the interviews was the complexity of antibiotic prescribing decisions. Some participants even stated that it was impossible to respond to interview questions about antibiotic prescribing for certain infections without additional information on the clinical scenario. One stewardship pharmacist champion highlighted the individuality of each patient’s situation and the difficulty of speaking in generalities about antibiotic prescribing: “The value of us [the stewardship team] reviewing all these antibiotics is that every single patient is slightly different and there’s lots of gray area and nuance.” A stewardship physician champion similarly stated, “The problem is that antibiotics are not black and white.” According to a different stewardship pharmacist, “That’s the theme of ID. Everything depends.”

**Table 1.** Characteristics of 15 Participating Hospitals in the Veterans’ Affairs—Centers for Disease Control and Prevention Practice–Based Research Network

Characteristic	No. (%)
**US Census region**	
Midwest	6 (40)
Northeast	1 (7)
South	5 (33)
West	3 (20)
**Hospital size**	
≥100 acute-care beds	10 (67)
<100 acute-care beds	5 (33)
**VHA hospital complexity level** ^a^	
1a	10 (67)
1b	4 (26)
1c	1 (7)
Antibiotic stewardship pharmacist and physician champions	15 (100)
Presence of clinical pathways and/or local antibiotic prescribing guidelines	7 (47)

a The Veterans’ Health Administration classifies its medical facilities at the following levels of complexity: 1a, 1b, 1c, 2, or 3. A hospital’s complexity level is based on its patient population, clinical services, education and research. The most complex hospitals are level 1a, and the least complex are level 3.

In total, 31 unique decisional factors were identified (Table 2) and were sorted into 1 of 5 categories: (1) type of infection, (2) patient-specific factors, (3) contextual factors, (4) provider-specific factors, and (5) drug-specific factors. Type of infection included factors like diagnostic test results, including microbiologic cultures, and the complexity of the infection. Patient-specific factors included age, comorbidities, and the patient’s risk of having an antibiotic-resistant infection. Contextual factors included local rates of antibiotic-resistant bacteria, social norms around antibiotic use, and antibiotic restrictions. Provider-specific factors included a provider’s knowledge, clinical instincts, and relative concern (or lack thereof) for antibiotic-related adverse outcomes. Drug-specific factors included antibiotic spectrum of activity, ease of dosing, and oral bioavailability. Participants reported considering many of these factors simultaneously when making prescribing decisions.

**Table 2.** Decisional Factor Categories and the 31 Decisional Factors That Influence Antibiotic Prescribing

Type of Infection	Patient-Specific Factors	Contextual Factors	Provider-Specific Factors	Drug-Specific Factors
Healthcare/hospital-acquired versus community-acquired	Age and comorbidities	Local rates of antibiotic resistant bacteria	Knowledge about the microbiology of the infection type	Spectrum of activity
Pathogens commonly involved in the infection type	Prior healthcare exposure	Antibiotic prescribing guidelines, both local and national	Preference for intravenous versus oral antibiotics	Bioavailability
Microbiology culture results from currently infected body site	Prior antibiotic exposure	Location and timing of patient-provider encounter	Clinical instinct informed by bedside assessment	Cost
Complicated vs. uncomplicated infection	Risk for an antibiotic-resistant infection, including prior microbiologic results	Ease of ordering the antibiotic, drug formulary, and prior approval processes	Desire to practice evidence-based clinical medicine	Ease of dosing
Depth/extent of infection	Renal function	Social norms around antibiotic use	Concern about antibiotic-related adverse outcomes in the patient	Frequency of antibiotic dosing
Severity of illness, including the presence of sepsis	Risk for developing *Clostridioides difficile*	Decisional support system	Concern about the societal problem of antibiotic resistance	…
Radiologic findings	Response to current antibiotic therapy	…	…	…

Some decisional factors that participants described were not well defined, such as “complicated infection,” “healthcare exposure,” or “septic.” For these terms, specific definitions were often not provided. As an example, the term “healthcare exposure” was frequently used to justify broader antibiotic therapy. A minority of participants expressed concern about this term, particularly in relation to pneumonia. According to one physician champion, “Studies have shown that the criteria or the scores to say this is ‘healthcare-associated pneumonia’ are no better than flipping a coin to identify somebody who has one of those resistant pathogens.” Instead of using the term “healthcare exposure,” a stewardship pharmacist champion explained that “we would try to do more risk factor-based therapy,” which would involve assessing specific risk factors for *Pseudomonas aeruginosa* or methicillin-resistant *Staphylococcus aureus*.

## Discussion

Our investigation of decisional factors revealed that providers consider >30 factors when making antibiotic prescribing decisions in acute-care settings. The influence of specific factors may differ across providers, but there was strong consensus that these prescribing decisions are complex.

Although many of the identified factors may seem obvious to clinicians who prescribe antibiotics, few studies have tried to broadly identify the multiple facets of these decisions, as we have done. Some of the decisional factors we identified have also been described in prior research about antibiotic decision making in studies across different types of infections, different countries, and different healthcare settings.^6–10^

Our findings may help identify potential targets for educating prescribers about antibiotics. For example, some participants stated a preference for using oral antibiotics with high bioavailability or for using intravenous agents over oral agents, and this finding highlights an opportunity for antibiotic stewardship teams to better define when these pharmacologic properties are most clinically relevant. Although healthcare exposure was often cited as a reason to prescribe more broad-spectrum antibiotics, the nuances of which types of exposure truly increase the risk of antibiotic resistance may not be as well understood.

Our study had several limitations. Although we enrolled a geographically diverse sample of providers, our findings may not be generalizable to non-VHA settings. Our interview guides were not created explicitly to parse out specific decisional factors. However, we would expect that if a decisional factor was relevant, it would have been raised by at least some of the 49 participants. We were able to identify decisional factors, but we were unable to assess the perceived importance of each of these factors in general and relative to different types of infections. To evaluate how participants prioritize these factors, our findings could be leveraged in future surveys and/or in-depth semistructured interviews that are specifically designed for this purpose.

In summary, we have elucidated many factors that providers consider when making antibiotic prescribing decisions for common infections in acute-care settings. This information should be considered when developing and implementing antibiotic stewardship strategies, particularly strategies focused on education.

## Supplementary material

For supplementary material accompanying this paper visit https://doi.org/10.1017/ash.2022.230. click here to view supplementary material
