# Supplementary material for: Investigation of factors influencing inpatient antibiotic prescribing decisions in the Veterans’ Health Administration
Source: Antimicrob Steward Healthc Epidemiol. 2022 Jun 23;2(1):e99. doi: 10.1017/ash.2022.230 (PMC9726494; doi:10.1017/ash.2022.230)
Supplement: Supplementary file 1 [file S2732494X22002303sup001.docx]

Interview Guide for Key Stakeholders

START with demographic questions sheet INTERVIEW

1. What is your role at this facility? (Hospitalist, Intensivist, or ED provider) How much time do you spend providing direct patient care?
2. What antibiotic do you usually initiate for the following conditions?
   1. What antibiotic do you usually initiate for **Community acquired pneumonia (CAP) in an immunocompetent patient?**
      1. What **oral antibiotic** do you prefer upon hospitalization or discharge?
      2. What is the role of 3rd or 4th generation cephalosporins (i.e., cefotaxime, ceftriaxone and cefepime) in the treatment of these infections?
      3. What is the role of fluoroquinolones (i.e., levofloxacin, ciprofloxacin, and moxifloxacin) in the treatment of these infections?
   2. What antibiotic do you usually initiate for **Community acquired UTI**?
      1. What **oral antibiotic** do you prefer upon hospitalization or discharge?
      2. What is the role of 3rd or 4th generation cephalosporins (i.e., cefotaxime, ceftriaxone and cefepime) in the treatment of these infections?
      3. What is the role of fluoroquinolones (i.e., levofloxacin, ciprofloxacin, and moxifloxacin) in the treatment of these infections?
   3. What antibiotic do you usually initiate for **Community acquired Skin/soft tissue infections**?
      1. What is the role of 3rd or 4th generation cephalosporins (i.e., cefotaxime, ceftriaxone and cefepime) in the treatment of these infections?
      2. What is the role of fluoroquinolones (i.e., levofloxacin, ciprofloxacin, and moxifloxacin) in the treatment of these infections?
   4. What antibiotic do you usually initiate for **Community acquired Intra-abdominal infections**?
      1. What is the role of 3rd or 4th generation cephalosporins (i.e., cefotaxime, ceftriaxone and cefepime) in the treatment of these infections?
      2. What is the role of fluoroquinolones (i.e., levofloxacin, ciprofloxacin, and moxifloxacin) in the treatment of these infections?
3. What **benefits** do you think 3rd or 4th generation **cephalosporins** offer over other antibiotic classes?
4. What **concerns** do you have, if any, when you prescribe 3rd or 4th generation **cephalosporins**?
5. What **benefits** do you think **fluoroquinolones** offer over other antibiotic classes?
6. What **concerns** do you have, if any, when you prescribe **fluoroquinolones**?
7. In general, would you say you are **satisfied with your access** to prescribe or recommend 3rd or 4th generation

**cephalosporins** and with therapeutic alternatives to these agents?

- 1. What agents do you typically prescribe in place of an a 3rd or 4th generation cephalosporin?

1. In general, would you say you are **satisfied with your access** to prescribe **fluoroquinolones** and with therapeutic alternatives to these agents?
   1. What agents do you typically prescribe in place of a fluoroquinolone?
2. Would you say that individuals **over prescribe or under-prescribe** 3rd or 4th generation **cephalosporins** at your facility? Why?
   1. What do you think are the most important changes to make to **reduce inappropriate use** of 3rd or 4th generation cephalosporins?
3. Would you say that individuals **over prescribe or under-prescribe fluoroquinolones** at your facility? Why?
   1. What do you think are the most important changes to make to **reduce inappropriate use** of

**fluoroquinolones**?

1. Tell me about **formulary restrictions** for 3rd or 4th generation cephalosporin and/or fluoroquinolones at your facility. What is the purpose or focus of these formulary restrictions?
   1. How do the restrictions impact work flow? (How practical are these restrictions?)
   2. [If audit and feedback isn’t specifically mentioned:] Does your facility use **audit and feedback** for 3rd or 4th generation cephalosporin and/or fluoroquinolones monitoring?
2. Does your facility have **guidelines or policies to promote reduction** in the use of fluoroquinolones or 3rd or 4th generation cephalosporins?
   1. If yes, what did these guidelines or policies say?
   2. How closely would you say individuals at your facility followed these guidelines or policies for the use of 3rd or 4th generation cephalosporins? For fluoroquinolones?
   3. **Do the policies in place** at your institution that restrict or guide the prescribing of 3rd or 4th generation cephalosporins **reduce inappropriate use** of these agents? For fluoroquinolones?
3. How does [or how would] your facility’s culture affect the success of ***prior approval*** for 3rd or 4th generation cephalosporin and/or fluoroquinolones?
4. How does [or how would] your facility’s culture affect the success of ***prospective audit and feedback*** for 3rd or 4th generation cephalosporin and/or fluoroquinolones?
5. *16. 17. OPTIONAL AS TIME ALLOWS*
6. Other than prior approval or prospective audit and feedback, have **other stewardship strategies** been implemented to improve 3rd or 4th generation cephalosporin and fluoroquinolones prescribing? Could you tell me about those?
7. What types of **educational programs**, if any, are offered at your facility related to antimicrobial prescribing?
   1. How are they offered or what format are they offered in? Grand rounds? Webinars? Conferences or in- person training?
   2. How frequently would you say these opportunities are offered? Is this often enough or should they be offered more frequently?
8. Outside of the VA, what **other programs or policies** are you aware of related to the reduction of 3rd or 4th generation cephalosporin and/or fluoroquinolone use?

*ASK FOR ALL*

1. What remaining thoughts do you have that you’d like to say at this time?

*Thank you for your participation in the study and in this interview, your thoughts will provide valuable information for larger efforts related to antimicrobial stewardship in VA.*
